# Surface Characteristics of One-Sided Charred Beech Wood

**DOI:** 10.3390/polym13101551

**Published:** 2021-05-12

**Authors:** Dita Machová, Anna Oberle, Lucie Zárybnická, Jakub Dohnal, Vít Šeda, Jakub Dömény, Veronika Vacenovská, Michal Kloiber, Jan Pěnčík, Jan Tippner, Petr Čermák

**Affiliations:** 1 Department of Materials and Research, Institute of Theoretical and Applied Mechanics of the Czech Academy of Sciences, Centre Telč, Prosecká 809/76, 190 00 Prague, Czech Republic; zarybnicka@itam.cas.cz (L.Z.); kloiber@itam.cas.cz (M.K.); 2 Department of Wood Science and Technology, Faculty of Forestry and Wood Technology, Mendel University in Brno, Zemědělská 3, 613 00 Brno, Czech Republic; anna.oberle@mendelu.cz (A.O.); vit.seda@mendelu.cz (V.Š.); jakub.domeny@mendelu.cz (J.D.); jan.tippner@mendelu.cz (J.T.); petr.cermak.und@mendelu.cz (P.Č.); 3 Faculty of Civil Engineering, Institute of Building Structures, Brno University of Technology, Veveří 331/95, 602 00 Brno, Czech Republic; dohnal.j@fce.vutbr.cz (J.D.); veronika.vacenovska@vut.cz (V.V.); pencik.j@vutbr.cz (J.P.)

**Keywords:** surface charring, wood modification, colour changes, surface roughness, fire resistance, phenol sulphuric acid assay, decay resistance, beech

## Abstract

The aim of this paper was to analyze selected properties of beech wood (*Fagus sylvatica* L.) treated by one-sided surface charring. Specimens were one-side charred with a hot plate using several time-temperature combinations (from 200 to 400 °C). Characteristics such as colour, discoloration, surface roughness, fire resistance, total carbohydrate content at several wood layers and decay resistance were evaluated. Surface charring was applied to the radial and tangential surfaces. Colour measurements showed that the surface of the wood turned grey due to charring. In addition to colour measurements, other experiments showed significant differences between radial and tangential specimens due to their different structures. The higher the temperature used in treating them, the lower the roughness values for radial specimens, while the trend for tangential specimens was the opposite. A smoother surface is more fire resistant, so radial specimens are generally better in this regard. Tangential specimens are more susceptible during preparation to forming cracks that impair flame resistance because a continuous protective densified layer is not formed. The determination of total carbohydrates revealed significant changes at various wood depths after surface charring. These changes were more predictable in radial specimens due to the annual ring orientation, because each layer consisted of a similar earlywood/latewood ratio. Finally, when decay resistance was assessed, weight loss was found to be lower in all specimens than in the references. The results suggest that charring at a particular combination of temperature and time improved the investigated properties of the surface-modified beech.

## 1. Introduction

The method of preserving the surfaces of wood by charring was probably unknowingly used in history when people learned to control fire. This is evidenced by archaeological finds of wooden sticks and spears that were deliberately charred [1,2,3]. There is no doubt that traditional charring is one of the oldest ways to increase the natural durability of wood by providing a brittle dark black charred surface layer. In Japan, the technique is called shou sugi ban—literally “burnt cedar wood”, a process used for exterior cladding and fencing [4,5].

One of the first scientific experiments on the verification of the wood charring effectiveness was carried out by Graham and Miller in 1957 on fence posts [6]. According to their results, the technique was in preserving wood from decay not as effective as the application of banned or restricted products, such as creosote and other hazardous compounds. Difficulties with controlling the source of heat and creating a uniform layer have led to modifications to the charring method. A practical experiment in 2019, based on traditional Japanese practice, proved that a protective effect could be achieved, but it has been suggested that this cannot be done ecologically, due to the large gas consumption required for the surface charring [7].

A contact heating system using a hot plate allows greater control of the conditions and begins to imitate ancient technology by creating a uniform surface. Kymäläinen et al. [8] described charring by using a hot plate at a high temperature, and applying a weight on top to avoid structural deformation. Their study proved that the protective properties of charring were influenced by the wood species (spruce and pine), the temperature and time of charring, and the evenness of treatment. The contact angle increased due to charring but the water uptake decreased only in spruce sapwood; pine did not experience the same effect. The study was followed up by work on the sorption characteristics of the charred surface, and their research proved that charring notably reduced liquid water and water vapour sorption, and reduced hygroscopicity, while chemical and dimensional stability depended on heating time and temperatures [9]. Čermák et al. [10] studied the characteristics of wood charred at 220 °C for 15 and 40 min. They proved that charring improved moisture-related characteristics and led to better mechanical properties. These three articles are the only ones that have addressed the issue of one-sided charred wood.

While the process of thermally modifying wood alters the physical properties of the whole piece of timber, charring only treats the surface. Therefore, a charred layer of wood forms on the surface and the characteristics of the wood beneath remain unchanged. In chemical terminology, this process is sometimes called pyrolysis, which is the decomposition of complex substances to simpler ones by heating. Pyrolysis is a kind of thermolysis, and is one of the processes involved in wood charring. Pyrolysis is typically simplified to “charring” for structural applications. Unlike combustion, pyrolysis refers to the thermal decomposition of a substance, is endothermic and can occur without an oxidising agent [11].

Wood reaches a high temperature during the charring process; high surface temperatures cause both chemical and physical degradation. Wood undergoes drying by evaporation of bound water at temperatures of 40–100 °C [12] while moderate depolymerisation reactions are involved but no significant decrease in carbohydrates occurs [13]. Chemical bonds begin to break at temperatures above 100 °C and the weight loss of carbohydrates and lignin of various types increases with temperature [14]. Thermal degradation between 100 and 200 °C is accompanied by emissions of carbon dioxide, water, methanol, formic acid and acetic acid, often leaving non-volatile decomposition products in the wood [15], dehydration and very “slow pyrolysis” start [11]. With prolonged exposure at higher temperature, wood can become charred. From this point on, we can observe exothermic oxidation reactions, glowing and flame. Exothermic oxidation reactions occur because ambient air can diffuse into and react with the developing porous char residue [14]. Thermal treatment takes place in the temperature range 160–260 °C [16], which approximates to the temperature at which water is split from the cellulose structure (150–240 °C) [17].

Generally, “significant pyrolysis” begins between 200 and 300 °C. Processes at temperatures above 200 °C are performed with the main intention not to dry the wood, but to achieve new material properties. In this range, the same gases are released as in slow pyrolysis, but with significantly less water vapour and some carbon monoxide with high-boiling-point tar, reactions are endothermic, and the products are almost entirely non-flammable [18,19]. Dehydration around 200 °C is primarily responsible for the pyrolysis of hemicelluloses (first breaking down at 200–260 °C) [17] and lignin (primary pyrolysis of lignin starts at 200–400 °C) [20]. Lignin decomposes across a wider temperature range than hemicelluloses and cellulose and, being the most stable of all the structural components, does not completely decompose pyrolytically until temperatures up to 700 °C are reached [21].

A third temperature regime or “rapid pyrolysis” takes place up to 500 °C [14]. Around 300 °C aliphatic side chains start splitting off from aromatic rings in the lignin; the reaction is exothermic, leading to secondary reactions among the products [22]. While in the range 300–350 °C a significant depolymerisation of cellulose is observable, from 300 to 450 °C flammable volatiles are vigorously produced. The products are largely combustible and include highly flammable tars in the form of smoke particles [18]. All these processes vary with the type of wood, density, time of heating etc. The ignition temperature of beech is around 400 °C [23,24], therefore, the highest temperature used in this experiment was 400 °C.

The aim of the research was to provide a comprehensive study of the properties of one-sided surface charred beech wood as the most heavily logged broadleaf species in the forests of the Czech Republic. Beech wood is a traditional material for wood-processing industry but it is not suitable for permanent exterior use due to its low natural durability and dimensional stability. The results should contribute to increasing the usability and competitiveness of beech wood by providing a sustainable material with improved performance. The novelty of this research can be seen in the following, (1) utilization of beech as a low durability wood species, which has not been widely tested this way yet, (2) testing of a wide range of temperature-time regimes, and thus providing a wide range of possibilities for future products (color variations, properties improvements) and (3) use of contact heating and temperature up to 400 °C, which allows a solid charred surface to be provided and offers an advantage over the traditional method leading rather to brittle surface.

## 2. Materials and Methods

### 2.1. Specimen Preparation

We studied European beech (*Fagus sylvatica* L.) wood obtained from a forest enterprise in Moravia, a region of the Czech Republic. The radial and tangential specimens of dimensions 50 × 20 × 300 mm (*R/T* × *T/R*× *L*) without defects were cut from a single board with the growth rings parallel to the edges. The radial and tangential specimens were sorted into six groups with the same average oven-dry density (689 kg/m^3^), representing the orientation of the charred surface regarding heat flow, temperature and time exposed (Table 1). Each group consisted of 10 specimens.

Specimens were charred on one side at temperatures of 200 °C, 250 °C, 300 °C, 350 °C and 400 °C at atmospheric pressure for 20, 6, 4, 2, 1 and 0.5 min, using a contact heating system with a hot plate (Ceran 500, Gestigkeit GmbH, Germany). Temperature-time regimes were selected according to pre-tests. The temperature control system was regulated by an electronic NiCr-Ni temperature sensor (regulation between 50 and 500 °C). Specimens were oven-dried to 0% moisture content (MC) (103 ± 2 °C for 24 h) before the surface charring. Specimens of three dimensions were cut from the charred pieces (40 × 20 × 50 mm for colour and roughness measurements, 40 × 20 × 20 mm for total carbohydrate determination and 25 × 20 × 50 mm for the wood decay test).

### 2.2. Microscopic Measurements—Colour and Roughness

The specimens were scanned using a VHX-6000 (Keyence, Itasca, USA) optical microscope with a VHX-S600E free-angle observation system (Z motorised). The lamp was part of the microscope and the distance of the objective from the specimen was the same for all specimens. The images were analysed using Photoshop software (Adobe Photoshop CS6) to get the values of L*, a* and b* and the colour change was quantified using a three-dimensional colorimetric system which was established by the International Commission on Illumination (CIE). The coordinates represent the lightness of the colour (from L* = 0 black to L* = 100 diffuse white), its position between red and green (a*, negative values indicate green, positive values indicate red) and its position between yellow and blue (b*, negative values indicate blue, positive values indicate yellow). Each set contained five specimens, the final values being the average of these values. The colour change (∆E*) was calculated from the differences between the reference and result values of colour parameters ∆L*, ∆a* and ∆b* according to the equation:

$$\Delta E^{*}=\sqrt{{\left(\Delta L^{*}\right)}^{2}+{\left(\Delta a^{*}\right)}^{2}+{\left(\Delta b^{*}\right)}^{2}}$$

Specimens were also characterised in terms of surface quality under the same microscope. Two parameters were determined during surface roughness testing (the profile roughness parameter and the area roughness parameter). Both parameters are the arithmetic average of the absolute deviation from the mean height of the surface but have some fundamental differences due to the use of profile or area [25]. In our case, we determined the area roughness parameters (*S_z_*, *S_a_*) which give more significant result values for the surface’s characteristics. *S_z_* is defined as the sum of the largest peak height value and the largest pit depth value within the defined area 20 × 20 mm. *S_a_* is the extension of the arithmetical mean height of a line to a surface. It expresses, as an absolute value, the difference in each point’s height compared to the arithmetical mean of the surface. This parameter was used generally to evaluate surface roughness.

### 2.3. Fire Resistance Test

The fire resistance was tested as a specimen’s weight loss during a five-min exposure to a naked flame. A special box was made for the measurement, the device being designed to provide uniform conditions for burning and determining weight loss (Figure S1). The box was connected to the smoke transfer to the fume cupboard, the drain was high enough for the direction of the flame not to be affected. The device included an analytical scale. A 30 mm-thick cement fibre board was used for the measurement, with a hole in the middle of the board that was 5 mm smaller than the size of the specimens. A Bunsen burner was used as a flame generator, burning propane. The height of the specimen placed on the board was adjusted so that the top of the flame was directly in contact with the specimen. The specimens were conditioned to the humidity of 10 ± 2% prior to the experiment. Specimens were placed on the fibre board connected to analytical scales that automatically collected data on weight loss during burning. Measurements were taken up to the point of burning through the specimen’s area; on average this took five minutes. The weight was automatically recorded at the point of equilibrium, so the time interval between measured values was different. Therefore, it was necessary to perform data interpolation. As above, each series contained five specimens, and the results are presented as average values.

### 2.4. Total Carbohydrate Content

An analysis of the total carbohydrate content was carried out to determine the severity of the surface charring and to what depth each group was adjusted. Charred specimens were cut with a microtome (Leica RM2125 RTS, Nussloch, Germany) into layers starting from the treated surface. In sum, 12 layers of 0.5 mm depth were cut from each specimen. Laser granulometer (Cilas LD 1090, Orleans, France) measurements were carried out to determine optimal grinding conditions. The particle size after specimens were mixed for different times (3 and 30 min at the same 30 oscillations per minute) was tested in water. Consequently, thermal analyses were performed to evaluate the effect of possible thermal degradation during specimen mixing. Analysis was performed using a thermal analysis instrument (STA 504, TA instruments, Wetzlar, Germany). Two specimens from the reference series (the first was milled for 30 min at 30 oscillations per minute, the second was chipped and unmodified from the reference) were placed in Al specimen holders and analysed in a nitrogen atmosphere at a heating rate of 20 °C/min in the temperature range 25–450 °C. The specimen weight ranged from 5 to 10 mg.

After determining the optimal milling conditions, specimens were prepared according to the same procedure. Collected layers were milled for 30 min in a Mini-Mill Pulverisette 23 (Fritsch, Idar-Oberstein, Germany) at 30 oscillations per minute. To reduce variability within the group, five specimens were mixed together while considering the same charring coding and the corresponding specimen layer position.

The powders obtained were further used for the determination of total carbohydrates according to the spectrophotometric method described by Čermák et al. [10]. Thus, 100 mg of wood powder was extracted with 5 mL of aqueous methanol (Penta Chemicals, Prague, Czech Republic) that had been volumetrically diluted to 50%. The extraction was performed at room temperature (around 23 °C). Tubes containing the specimens and methanol were shaken for 15 min at 100 rpm (orbital shaker Miulab, Hangzhou, China), then sonicated for 15 min (ultrasound bath GT Sonic, Meizhou, China) and, finally, shaken again for 15 min [10]. After the extraction, specimens were filtered and stored at 4–8 °C until analysis. All fresh extracts were subjected to analysis within 48 h.

Phenol (Penta Chemicals, Czech Republic) was dissolved in demineralised water to a concentration of 5 wt.%. An aliquot 500 μL of the extract was filled with demineralised water to produce a 2 mL extract specimen solution, to which 1 mL of 5 wt.% phenol solution was added and vortexed; after the immediate addition of 5 mL of concentrated sulphuric acid (Penta Chemicals, Czech Republic) the mixture was stirred moderately and afterwards kept at room temperature for 10 min [10] to cool. The mixture was vortexed, then kept in a water bath at 30 °C for another 20 min, followed by a further 30 min at room temperature [10].

An ultraviolet–visible (UV-Vis) spectrophotometer (Metash, Shanghai, China) was used for all measurements. The absorbance of each specimen was recorded at a wavelength of 490 nm with subtracted absorbance of the solvent (demineralised water). The response of the standard solution of D-glucose (Sigma-Aldrich, Prague, Czech Republic) in the concentration range of 12.5–125 μg GluE/mL, after the reaction with phenol-sulphuric acid, was plotted as a linear calibration curve (R^2^ ≥ 0.9927 for all calibration cases). Thus, the final amount of saccharides present in the layer was based on the standard calibration expressed in glucose equivalents per gram of wood specimen (mg GluE/g wood). Fresh phenol solution was prepared daily and used for the calibration that served for the saccharide content calculation of two sequential specimen series measured on the same day. One blank specimen from each series served as an error controller within the series. All measurements were taken in duplicate, i.e., in two independent, separated runs that included the second weighing and extraction step.

### 2.5. Decay Test

The decay test was performed according to the modified EN 113 standard [26]. The specimens were carbonised on one side only; for this reason the sides of the specimens were sealed with an epoxy resin. When the epoxy was cured, specimens were dried at 105 °C for 24 h and the dry weight was measured before testing. Wood specimens were exposed to two types of pure culture: the brown rot fungus *Coniophora puteana* (CP) and the white rot fungus *Trametes versicolor* (TV). All specimens were sterilised with a germicidal lamp in a laminar box (Mercy UCS 3, Brno, Czech Republic) and placed in a Kolle flask on a sterile culture medium (malt extract agar) previously exposed to fungi for a week. The specimens were placed on rectangular structures printed on a 3D printer (from polylactic acid and sterilised in an autoclave) so that they were not in immediate contact with the agar. The Kolle flasks were incubated for 16 weeks at 20 °C after which the mycelium was removed, and specimens were dried and reweighed. Each set contained five specimens, the final values being the average of these values and the standard deviation values calculated.

## 3. Results and Discussion

### 3.1. Colour Measurements

**Figure 1:**
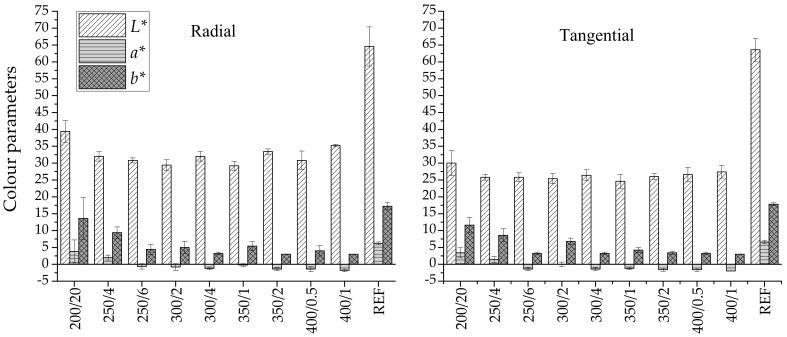
Colour parameters of radial and tangential specimens.

The colour space CIEL*a*b* of beech wood charred with different temperatures and times was investigated. Figure 1 shows the result of the colour measurement parameters L*, a*, b* for all specimens. Each set contained five specimens, and each specimen was measured five times; the arithmetic means of these measurements were calculated. Photos of treated beech are located in supplementary material (Figures S2 and S3).

Lightness (L*) or black-white relation is most affected by thermal treatment. The loss of lightness was approximately 50% in radial and 42% in tangential specimens (compared with the reference); and this was evident in all specimens. Darkening did not correlate with increasing charring or increasing time. Lightness usually decreases due to increasing temperature and time, and correlates with these factors [27,28]. However, in the case of our experiment, charring took place for minutes rather than hours, which led to the uniformity of lightness and colour changes taking place only on the outer surface layer. A decrease of L* means that the wood becomes darker. Hemicelluloses, especially pentosanes [29], are the components damaged most during heat treatment, thus a change in L* causes wood surface darkening. Consequently, the lignin content relative to hemicellulose of heat-treated wood increases accordingly [30].

The changes in the parameter a* in the radial and tangential direction were more or less the same, around 24%. The parameter a* became negative for temperatures of 250 °C and higher. The arithmetic mean for parameter b* was 33% for radial and 29% for tangential. At equivalent values of L*, the modification in b* was larger than in a*; in general, a reduction in a* and b* was affected by increasing temperature and time. A decrease in a* values indicates a tendency for the surface to become greener, and a decrease in b* indicates a tendency to become bluer, after treatment. An increase in a* and b* (red and yellow substances are being produced) is more typical due to rising temperatures. This is largely influenced by the type of wood, its chemical composition and the length and method of heating [28]. When the chromatic values decrease to zero or almost zero, the wood specimens become essentially hueless. The colour evolution is, therefore, simply explained as darkening towards dark grey. Grey colours are located in the centre of a* and b*.

**Figure 2:**
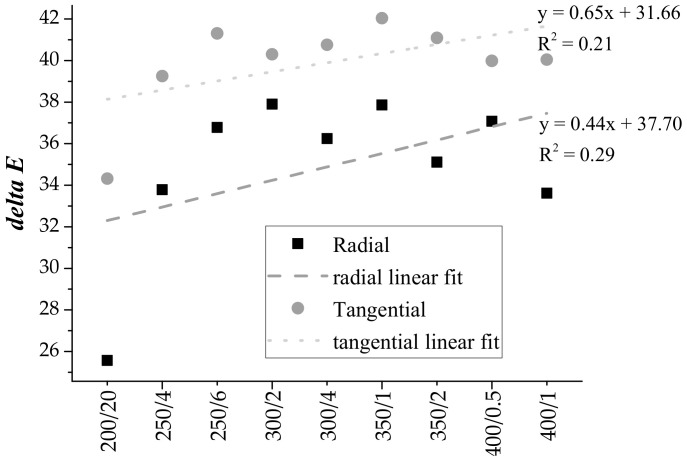
Comparison of colour changes ΔE in radial and tangential specimens.

The colour change ∆E* was highly influenced by L* (~50% decrease). The nature of ∆E* is a complex process as all wood components including the extractives may contribute to the change. Substantially, it can be seen from Figure 2 that the ∆E* of the tangential specimens was significantly smaller than the radial ones. This is also confirmed by the results of the change of colour parameters, which were smaller in the tangential direction. Tangential specimens in this sense had better colour stability. The linear fit shows an upward trend in both: the higher the temperature, the greater ∆E*. The fitted points and the associated values of R suggest a stronger relationship between temperature and ∆E* for tangential specimens (R = 0.54) than for radial ones (R = 0.46). However, the results also suggest that colour is not an unambiguous indicator of the degree of charring. For example, the ∆E* of radial specimens treated at 300 °C shows a greater value for those that were charred for two minutes than for those that were charred for four minutes (the same for 350/1_R_ and 350/2_R_, 400/05_R_ and 400/1_R_, 350/1_T_ and 350/2_T_). It appears that the temperature affects ∆E* more than the duration of the treatment.

### 3.2. Results of Surface Roughness

Roughness as another important indicator of surface quality is presented in Table 2. The results indicate that radial roughness decreases with increasing temperature and time. The lowest values of roughness were shown by the specimens 350/1_R_ and 350/2_R_ with values *S_a_* 0.118 mm, *S_z_* 1.103 mm and *S_a_* 0.110 mm, *S_z_* 1.112 mm, respectively. In contrast, radial specimens at 400 °C had increased roughness values because the surface of the wood specimens had already been deformed by cracking, whereas the results for the tangential specimens did not yield such a clear conclusion. There is no noticeably significant effect from the temperature and time of the charring. Compared to the reference, a reduction in the value of the roughness of the specimen 400/1, where it was measured *S_a_* 0.117 mm, *S_z_* 1.135 mm and for reference *S_a_* 0.123 mm, *S_z_* 1.283 mm, can be observed. When radial and tangential specimens are compared after the thermal process, the radial specimens were found to be smoother and there was a more significant change compared to the reference.

A comparison with the reference radial and tangential specimens is shown in Figure S4. Negative values of the measure indicate how much smoother the surface is than the reference. Positive values of roughness indicate how much rougher the surface is compared to the reference. The results show that, with higher temperature, there was a decrease in roughness values for radial specimens. For tangential specimens, the trend was reversed. A similar effect was observed, for example, in specimens of Turkish hazel (*Corylus colurna* L.) wood at lower temperatures up to 180 °C in a study [31], in which the surface became smoother with increasing temperature. Very interesting results were that surface roughness decreased by nearly 37% in the specimen heat-treated at 180 °C for 10 h when compared with the control specimens. Temperatures above 160 °C had a significant effect on lignin and compacting the wood surface [32]. Similar conclusions can be drawn from this study [33].

Roughness measurements can represent the overall surface quality [34]. Wood surface roughness values are influenced by various factors such as annual ring width [35], age, the density, the content of specific cellular structures [36] and the size of the contact angle [37,38]. It can also be concluded that surface roughness affects other wood properties. The logical assumption is that a surface with less roughness will be more resistant to fire [39], water absorption [40] and decay [41]. Nonetheless, water absorption in beech is nearly the same in both directions, which justifies categorising beech in the semi-ring-porous group [42].

### 3.3. Fire Resistance Test Results

**Figure 3:**
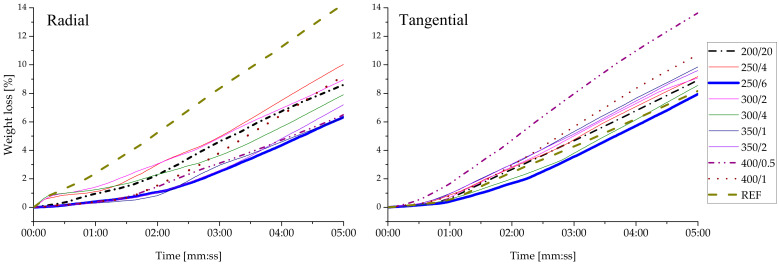
Weight loss of charred specimens recorded within five minutes in the fire resistance test.

As a consequence of pyrolysis, a charcoal layer is produced on the wood surface exposed to fire. The char layer is not resistant to mechanical force, but it has a protective effect for the interior of the wood specimens. For example, the char layer can play a substantial role in thermal insulation. Weight losses during combustion that lasted five minutes are recorded in Figure 3. The fire resistance test on radial specimens revealed a strong reduction of burning on charred specimens as compared to reference. The REF_R_ lost 16.6 wt.% (10.99 g) of its weight within five minutes, while the smallest weight loss was recorded for the specimen 250/6_R_ (6.8 wt.% = 4.17 g). Specimen 250/6_T_ was also found to be the most resistant to burning (8.6 wt.% = 5.72 g). Charring inhibits combustion more significantly in radial specimens. Tangential specimens 400/0.5_T_ and 200/20_T_ showed greater weight loss, their inhibition of combustion was worse; the rest exhibited an improvement in fire resistance.

Initially, weight loss is caused by loss of water. In most specimens, the initial change in weight is slight, as the specimens had been dried and conditioned to a humidity of 10 ± 2%. Polymer decomposition is indicated by weight loss starting at 200 °C when the hemicellulose component begins to decompose, until approximately 290 °C at which point a reduction of this rate is found. Above 290 °C the rate of weight change again increases, as the cellulose and lignin decompose, and reaches a maximum at 360 °C. The cellulose has largely decomposed at this temperature while the lignin components continue to break down [17]. A layer of char is formed as wood burns; charcoal is the last component to be burned, as it requires a higher temperature to ignite. This layer with its low thermal conductivity protects the wood and prevents further fire penetration [43]. The dense char layer creates an insulation barrier, reducing thermal and oxygen diffusion, which causes a reduction of the heat release rate and hinders the combustion reaction [44]. During combustion surface shrinks, which causes cracks that facilitate the passage of flammable gases to the surface [45]. Cracks in the char layer increase the heat transfer into the pyrolysis zone [46]. This explains why tangential specimens are more varied due to the orientation of the structural elements. There is a greater degree of crackling, which in turn leads to a deterioration of the fire protection. The flame resistance test was performed by White [47] on wood prepared by the shou sugi ban method, and no appreciable increase in fire resistance was found. It should be added that this test was performed on only one specimen with an undefined direction of preparation (radial/tangential) and under undefined conditions. As a rule, the less rough the specimen’s surface is due to rising temperature, the more fire-resistant it becomes. However, in our case this rule only applied to radial specimens.

### 3.4. Total Carbohydrate Content

The specimens were tested for thermal stability and particle size distribution before analysing the total carbohydrate content. This characterisation was necessary because the specimens were milled to ensure the required reproducibility. The thermal analysis did not show a negative effect of the selected time and mixing speed on the quality of the wood specimens (Figure S5). The difference between the values for the milled and chipped specimens was not significant. The longer grinding makes the powder more homogeneous, thus a 30 min grinding time was optimal because the measurement results over a longer period did not show differences in the size of the ground particles. The particle size of the milled specimens was D_10_ = 14.50 μm, D_50_ = 55.98 μm, D_90_ = 151.09 μm and mean diameter 71.59 μm.

**Figure 4:**
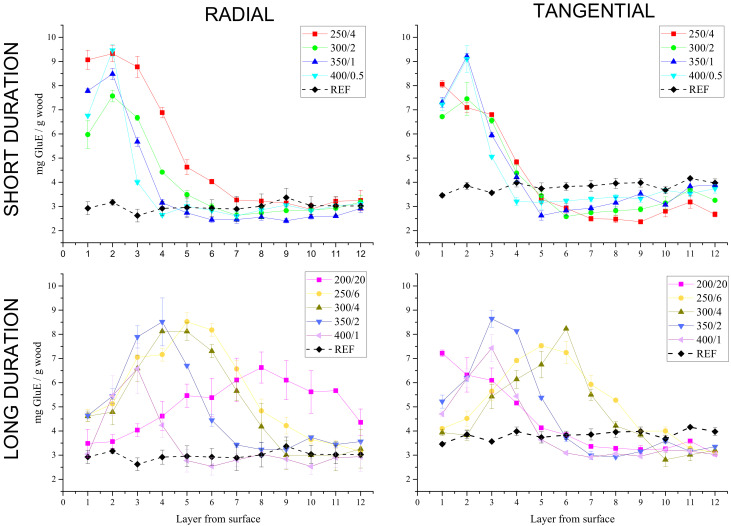
The effect of radial and tangential charring on the total carbohydrate content within 6 mm from wood surface (12 layers; each having 0.5 mm thickness).

Figure 4 illustrates the changes in the total carbohydrate content depending on the orientation of the wood (radial and tangential) and the temperature-time regimes applied for the one-sided surface charring. To better distinguish all charring regimes, radial and tangential treatments were used, respectively; the results are divided into four separated graphs based on (i) radial/tangential treatment direction, and (ii) short/long duration of the charring. The results from all charring groups can be seen within Figure 4.

While on the x-axis the numbers 1–12 represent the specific layer (each 0.5 mm thick) in rows from the charred surface, the y-axis represents the average amount of soluble carbohydrates detected in this particular layer. The Dubois assay [48] for total carbohydrate determination is applicable to both reducing and non-reducing saccharides and is feasible also for oligo- and polysaccharide quantification. For specimens containing glucose as the main structural unit, calibration is performed using glucose solution [49]. Pentosans and hexosans from hemicelluloses undergo in a hot and acid environment structural depolymerisation and dehydration to furfural and hydroxymethylfurfural [50,51,52], respectively. Furfural and hydroxymethylfurfural then react with phenol yielding yellow products with maximum absorbance at 490 nm [51]. Thanks to this shift from ultraviolet (UV) to the visible region of the spectrum, better sensitivity is achieved [51] and the accuracy for hours-stable formed compounds is under 2% [52]. Based on this analysis, it is possible to conclude at which depth each group has been modified and the charring process can be optimized accordingly.

Selecting short treatment durations led to uncertain differentiation of the changes in carbohydrates between the charring groups (250/4, 300/2, 350/1, 400/0.5; compare top graphs). In cases when the treatment duration was increased, the effect of applied temperature and anatomical orientation became more highlighted (bottom graphs).

The impact of charring conditions appeared almost identical for 350/1_T_ and 400/0.5_T_. Other groups of tangential treatment also did not differ much from each other within the specimen’s depth, with signs of modification up to the fourth layer (i.e., 2 mm from the surface). From the fifth layer, the trend for all briefly cured specimens (250/4_T_, 300/2_T_, 350/1_T_, 400/0.5_T_) is below the line of the reference specimens, indicating a shift of carbohydrates to the surface induced by charring.

The reference specimens were revealed to have a slightly different starting point for the charring process. Due to the different anatomical orientation of the wood and moreover varying annual ring proportions, REF_R_ had 2.99 mg/g GluE and REF_T_ 3.84 mg/g GluE, as the mean value of layers up to 6 mm depth. This aspect of varying ratio of earlywood and latewood was proved to increase the differences between the radial and tangential specimens, when prolonged charring settings were applied (the two bottom graphs). Based on the curves of the radial specimens (bottom left), such as 250/6_R_, then 300/4_R_, followed by 350/2_R_ and 400/1_R;_ a tendency of broadening the “peak” created at those layers, resulting in the highest carbohydrate amounts, can be observed. With decreasing temperature and conversely an increase in the reaction time for the heat treatment, a clear “peak” shifts its maximum to the right (meaning away from the surface).

The modification for 400 °C (400/1_R_ and the same for 400/1_T_) reached the first four layers (i.e., 2 mm from the charred surface). The other temperature-time groups were also affected in the deeper layers in the following order: 350/2_R_ (6 layers), 300/4_R_ (8 layers), 250/6_R_ (10 layers), 200/20_R_ (all 12 layers). The same applies to specimens charred for shorter durations: 400/0.5_R_ (3 layers), 350/1_R_ (4 layers), 300/2_R_ (5 layers), 250/4_R_ (6 layers), however those specimens exhibited a narrower distribution width with respect to charring time. Such a neat “distribution” might be related to the almost homogeneous structure, as the earlywood/latewood ratio in radial orientation remains constant, therefore the reactivity alters periodically with changing polymer composition in neighbouring layers.

It can be concluded that in radial orientation the time of charring had a more significant impact on the number of modified layers than the temperature. This was not the case for tangential specimens (bottom right). A similar effect was observed on beech charred at 220 °C for 15 min when radial specimens showed no significant difference from the non-charred reference within 2 mm from surface, whereas mean values for two layers (2 mm in total) of soluble carbohydrates of those with the tangential orientation were almost twice as high as in radial [10]. The reverse behaviour of 200/20_R_ (increasing tendency from first layer) and 200/20_T_ (the first layers showed the highest value) might be attributed to dehydration reactions still occurring [14].

### 3.5. Decay Resistance Test

As mentioned above, charred wood is probably more resistant to fungal attack than untreated wood because its chemical composition varies after surface charring. Wang et al. [53] assumed that the nutrients in wood are reduced due to thermal treatment to such an extent that it improves resistance to decay. The experimental conditions of this research group differed from ours, but the resistance of the wood correlated with increasing temperature and treatment duration. In this study, experiments were conducted with white and brown rot fungi. Fungi have evolved strategies to degrade wood using a range of enzymes and consume cell wall components for their nutrition [54]. Generally, three hypotheses have been formulated to explain the increase in decay resistance [55]: (a) the formation of toxic compounds during heating, (b) the low affinity of heat-treated wood for water and (c) the degradation and chemical modification of major wood polymers. For the fungal decay test, brown (CP) and white (TV) rot fungi were used. TV formed a dense mycelium film over the specimen, thereby helping to increase the moisture level; meanwhile CP created a low moisture content. Typically, the degradation of hemicelluloses is accompanied by the release of acetic acid. A high temperature and low pH give rise to the acid hydrolysis of polysaccharides and lignin. The bonds of saccharide units break and intramolecular dehydration reactions take place [56]. TV belongs to a group of simultaneous degraders, meaning that it degrades all cell-wall components simultaneously (cellulose, hemicelluloses and lignin). Brown rot fungi rapidly and extensively depolymerise cellulose and hemicelluloses, leaving behind lignin, which is only partially modified and the cell wall is transformed into a porous structure [57,58].

**Figure 5:**
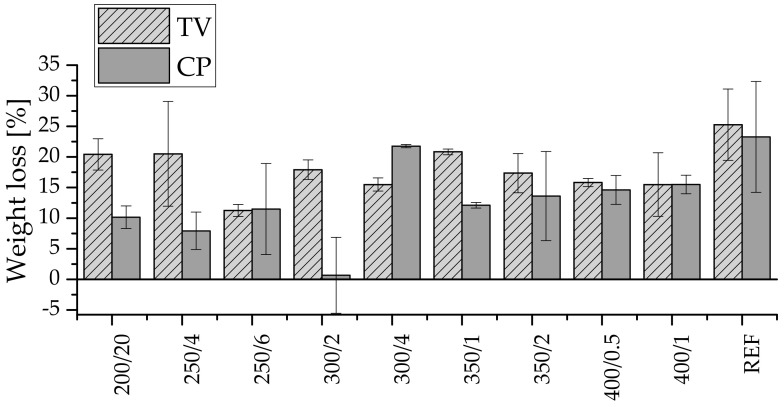
Weight loss of wood attacked by *Coniophora puteana* (CP) and *Trametes versicolor* (TV).

The protective treatment is effective when the weight loss after decay test is less than 3 wt.%, this result has only been recorded for sample 300/2 of wood attacked with CP (Figure 5). The results of the decay test revealed that there was less weight loss in wood attacked by CP (average 11.61 wt.%) than TV (average 18.04 wt.%). TV tends to be more aggressive on hardwoods such as beech, which was evident from our results, because charring did not prevent much weight loss compared with CP. An interesting finding is that while weight loss increased slightly in specimens attacked by TV, it decreased in the case of CP. In other words, increasing the temperature during the charring process reduced the resistance of beech to CP and increased the resistance to TV (Figure S6). Understanding the behaviour of rotting fungi in more depth will require knowledge of gene expression. However, the positive effect of charring on decay resistance has been demonstrated.

Indeed, the charring group 250/6 (both radial and tangential) behaved in several aspects differently from the other groups. White rot and brown rot fungus resistance was not as effective as it was supposed to be for a functional wood protection treatment; however, the weight loss was half that of the reference specimens, independent of the fungus (Figure 5). Fire resistance was proven to work better in all groups (Figure 3). That can be explained by the total carbohydrate content of the analysed layers (Figure 4). Distinct amounts of carbohydrate were detected in several layers, leaving the first millimetres with low content, increasing to a maximum at 2.5–3.0 mm from the surface and then slowly decreasing the thermal effects.

The anisotropic character of wood is characterised by different values in different directions, and in this case in two orientations (radial and tangential). Naturally, properties in the tangential and radial directions are assumed to differ. For instance, the values of dynamic and static bending strength of wood are different depending on the direction of the external force applied (in the radial or tangential loading direction). The difference of mean values in impact bending strength in the radial direction towards tangential was observed to be about 20% for beech [59]. Thermal conductivity of beech is about 18% higher in the radial than the tangential direction. Vay et al. [60] attributes this to the high number of ray cells in beech wood. Thermal conductivity is also strongly influenced by moisture and temperature [61].

## 4. Conclusions

The one-sided surface charring of beech wood (*Fagus sylvatica* L.) prepared by several time-temperature combinations was investigated to identify changes in selected properties. Colour measurements indicated that the lightness L* was more or less the same no matter what the temperature or time, apart from the parameters a* and b*, which decreased with rising temperature and time, making the wood darker grey. At higher temperatures, the roughness of the radial specimens decreased, while the trend was reversed for tangential specimens. The variety of our tangential specimens affected the results of the flame resistance test. All radial specimens had better fire resistance than the reference specimens, but for tangential specimens there was less weight loss only at a temperature of 300 °C and above. However, the tangential reference specimens exhibited much less weight loss than those of the radial reference. Beech treated at 250 °C for six minutes achieved the best result in both directions. Similarly, six minutes at 250 °C was the optimal treatment to modify the greatest thickness of wood in all groups (up to 4.5 mm from the surface) just after several minutes. Current research is mainly focused on understanding the thermal processes when a whole piece of wood is modified for hours. Therefore, no explanation is available for what happens to the surface in time/temperature dependency when only one side is charred. The phenol-sulphuric acid assay we used cannot be considered a suitable tool only for identifying the depth to which charred wood is modified, but more importantly, it could be used to optimise the charring process to create wood with desired properties. Radial orientation of specimens was found to be best suited for the temperature-time comparison studies. The only one specimens that had the resistance to the brown rot fungus were prepared at 300 °C for two minutes. There is no doubt that the properties of charred wood are largely influenced by its anatomical structure, which depends on the cut direction (radial, tangential).

The technology of one-sided charring opens up new possibilities for the use of wood for decorative purposes while offering fire and partial fungal protection. Moreover, reducing the energy consumption of the procedure (minutes instead of hours) gives this technology a “green” advantage over chemical treatments. Limits of one-sided charred beech wood for exterior use are found mainly in dimensional changes, where during the swelling of the wood the surface of the charred layer may cause cracks and thus its protective properties decrease, and also, for example, in use in fasteners, where the modified surface layer may be partly damaged.

## Figures and Tables

**Table 1:** Coding of specimens referring to the direction, temperature and time of charring.

Group	Direction	Temperature [°C]	Time [min]	Coding	Group	Direction	Temperature [°C]	Time [min]	Coding
REF	R	-	-	REF_R_	Charred specimens	R	300	4	300/4_R_
	T			REF_T_		T			300/4_T_
Charred specimens	R	200	20	200/20_R_		R	350	1	350/1_R_
	T			200/20_T_		T			350/1_T_
	R	250	4	250/4_R_		R	350	2	350/2_R_
	T			250/4_T_		T			350/2_T_
	R	250	6	250/6_R_		R	400	0.5	400/0.5_R_
	T			250/6_T_		T			400/0.5_T_
	R	300	2	300/2_R_		R	400	1	400/1_R_
	T			300/2_T_		T			400/1_T_

**Table 2:** Mean values of surface roughness and standard deviations for radial and tangential specimens.

Specimen	Radial		Tangential	
	*S_a_* [mm]	*S_z_* [mm]	*S_a_* [mm]	*S_z_* [mm]
200/20	0.201 (0.01)	1.179 (0.08)	0.142 (0.01)	1.260 (0.16)
250/4	0.146 (0.06)	1.410 (0.63)	0.122 (0.04)	1.349 (0.83)
250/6	0.137 (0.03)	1.370 (0.26)	0.156 (0.02)	1.454 (0.23)
300/2	0.118 (0.02)	1.168 (0.21)	0.132 (0.02)	1.424 (0.34)
300/4	0.136 (0.02)	1.365 (0.27)	0.171 (0.05)	1.479 (0.34)
350/1	0.118 (0.01)	1.103 (0.15)	0.128 (0.01)	1.394 (0.27)
350/2	0.110 (0.01)	1.112 (0.19)	0.130 (0.01)	1.306 (0.30)
400/0.5	0.135 (0.02)	1.262 (0.27)	0.116 (0.03)	1.204 (0.20)
400/1	0.133 (0.00)	1.189 (0.10)	0.117 (0.02)	1.135 (0.16)
REF	0.151 (0.07)	1.438 (0.03)	0.123 (0.00)	1.283 (0.24)

## Supplementary Materials

The following are available online at https://www.mdpi.com/article/10.3390/polym13101551/s1: Figure S1: Box for fire-resistance test; Figure S2: Charred specimens in the radial direction; Figure S3: Charred specimens in the tangential direction; Figure S4: Roughness results compared with the reference; Figure S5: Thermal analysis of milled and reference specimens; Figure S6: Comparison of weight loss of specimens attacked by *Trametes versicolor* and *Coniophora puteana*.
