# Supplementary material for: Surface Characteristics of One-Sided Charred Beech Wood
Source: Polymers (Basel). 2021 May 12;13(10):1551. doi: 10.3390/polym13101551 (PMC8151175; doi:10.3390/polym13101551)
Supplement: Supplementary file 1 [file polymers-13-01551-s001.zip › polymers-1204441-supplementary.pdf]

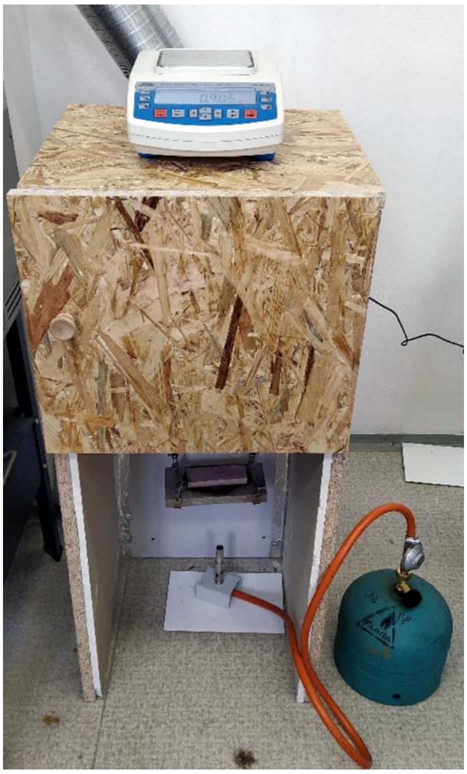

Figure S1 Box for fire-resistance test.

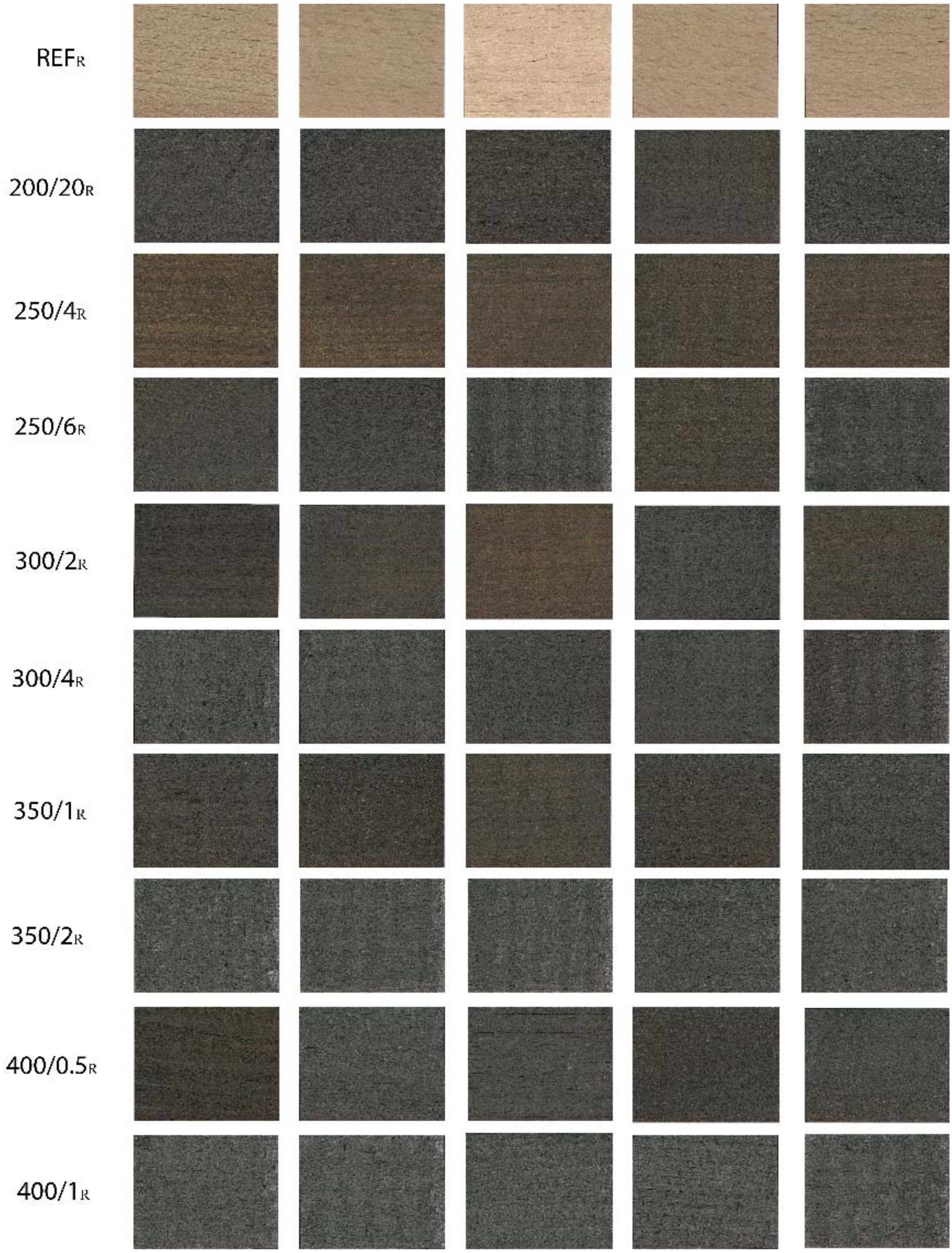

Figure S2 Charred specimens in the radial direction.

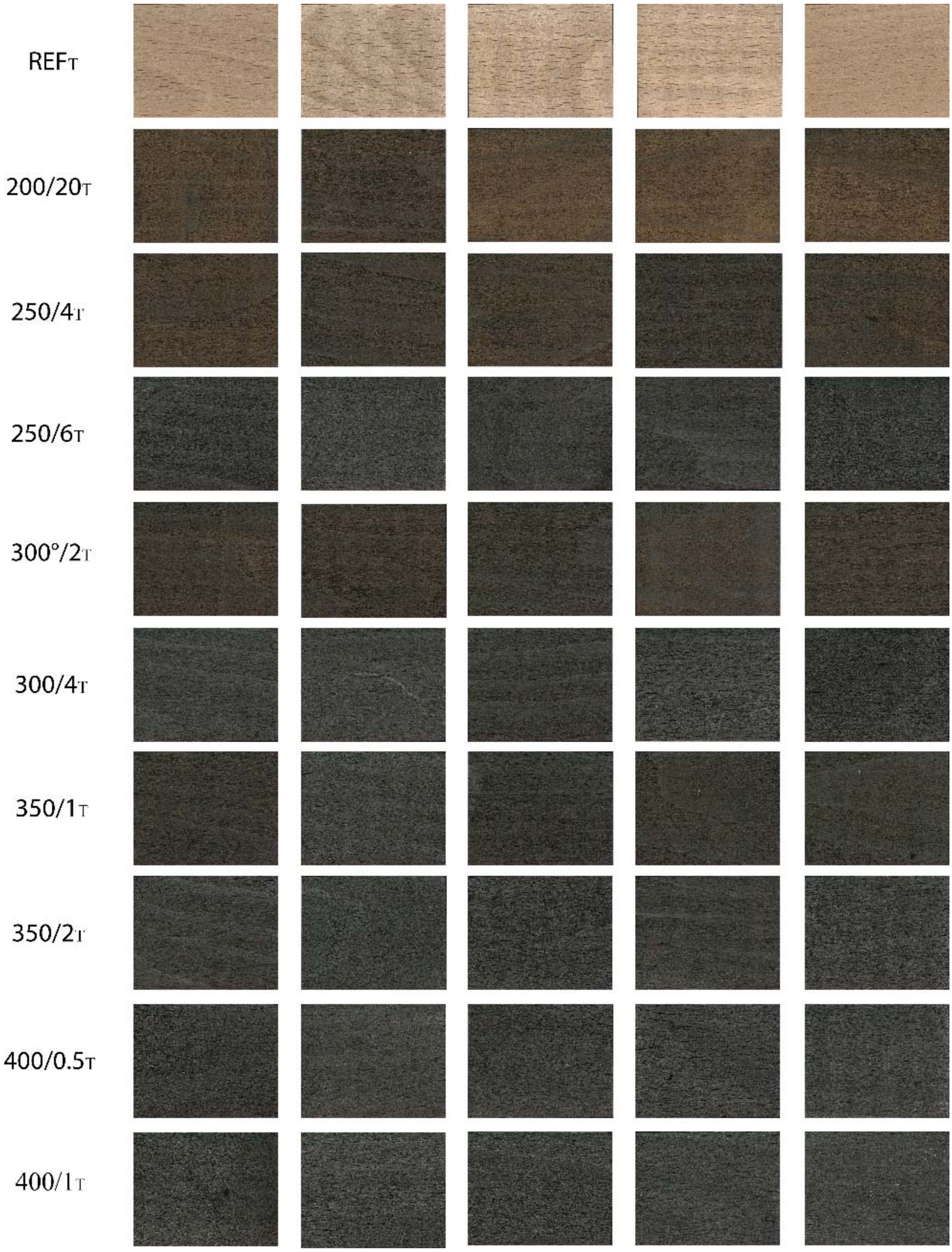

Figure S3 Charred specimens in the tangential direction.

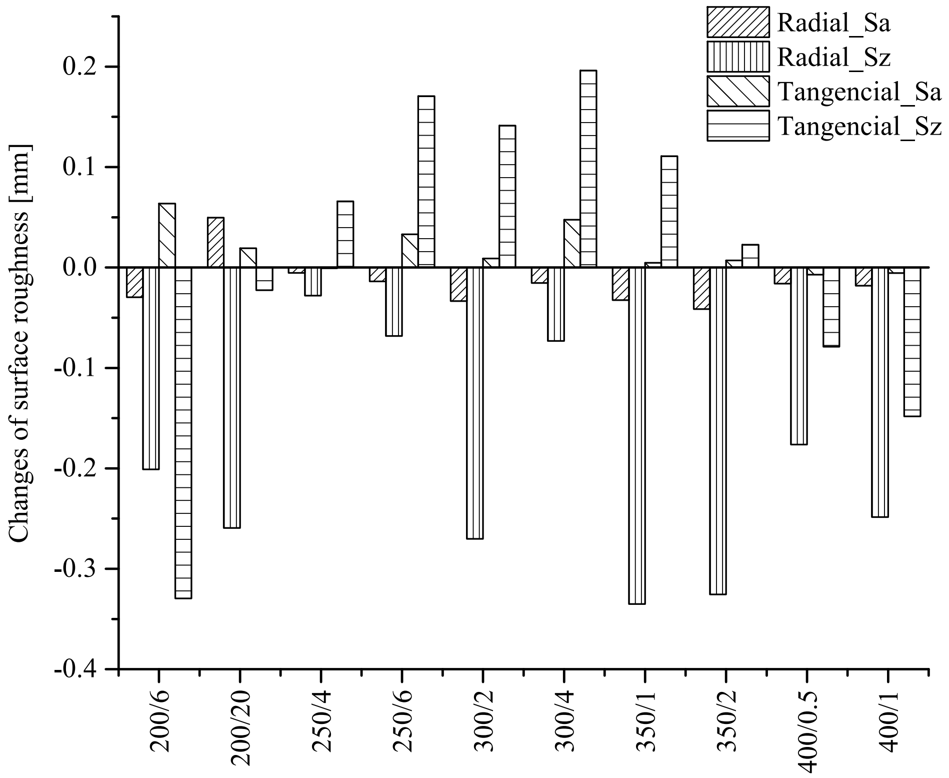

Figure S4 Roughness results compared with the reference.

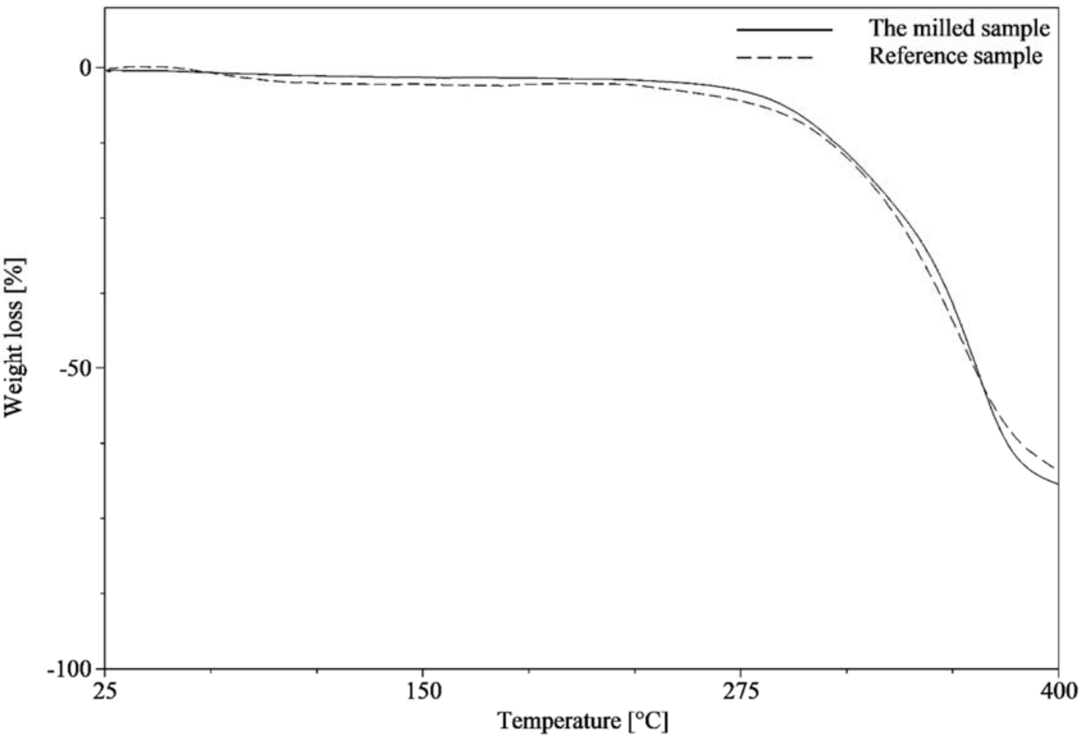

Figure S5 Thermal analysis of milled and reference specimens.

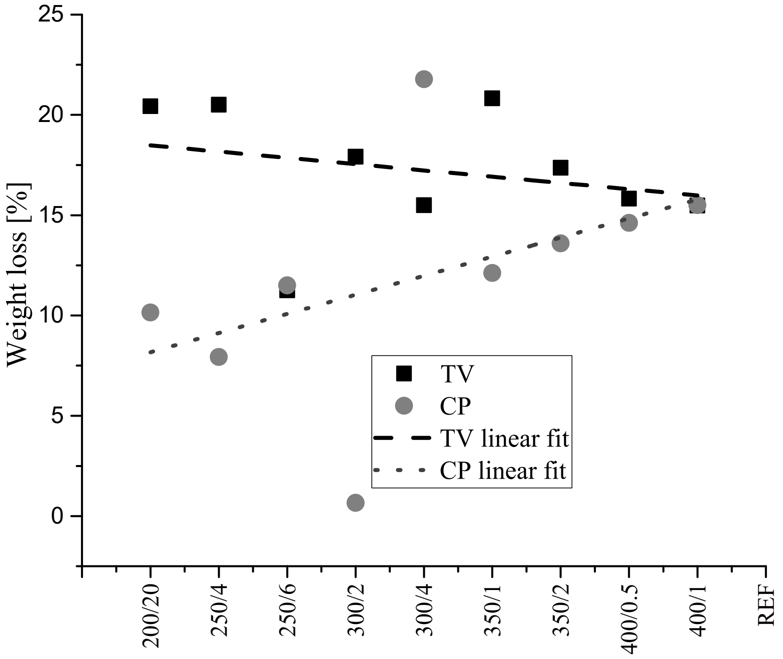

Figure S6 Comparison of weight loss of specimens attacked by Trametes versicolor and Coniophora puteana.
